# Tungurahualini, a new tribe of Neotropical leafhoppers, with notes on the subfamily Mileewinae (Hemiptera, Cicadellidae)

**DOI:** 10.3897/zookeys.124.1561

**Published:** 2011-08-18

**Authors:** C. H. Dietrich

**Affiliations:** Illinois Natural History Survey, University of Illinois, 1816 S. Oak St., Champaign, IL 61820, USA

**Keywords:** Homoptera, Auchenorrhyncha, morphology, identification, distribution

## Abstract

A new cicadellid tribe, Tungurahualini, is recognized to include *Tungurahuala* Kramer, and a related new genus, *Ilyapa* **gen. n.**, based on six new species. The tribe is included in subfamily Mileewinae, the concept of which is further expanded to include tribes Makilingiini Baker, and Tinteromini Godoy and Webb, taxa previously treated as separate subfamilies. Keys to tribes of Mileewinae (sensu lato) and genera of Tungurahualini are provided. A new species of *Tungurahuala*, *Tungurahuala acuminata* **sp. n.**, is also described and keys to species of *Tungurahuala* and *Ilyapa* are provided. The new tribe is presently recorded only from cloud forests in the northern Andes Mountains of South America.

## Introduction

The leafhopper subfamily Mileewinae comprises small to medium-sized, slender, usually darkly pigmented species that inhabit wet tropical forests worldwide. Most species appear to inhabit montane cloud forests where they occur on herbaceous vegetation in the understory. The group was established by Evans (1947) as a tribe of Cicadellinae (as “Tettigellinae") based on the dorsal ocelli, narrow gena, and strongly convex frontoclypeus. Evans distinguished the tribe from other Cicadellinae based on the forewing “with a reduced clavus, a wide appendix and lacking vein M1+2 [= r-m1]." Young (1965) transferred the tribe from Cicadellinae to Typhlocybinae because he considered Mileewini to have “much more in common" with the latter subfamily, but did not mention particular characters that supported this new placement. In his subsequent comprehensive revision of world Cicadellinae, Young (1968) initially continued to treat Mileewini as a tribe of Typhlocybinae but later (Young 1986) followed Linnavuori and DeLong (1977) who elevated Mileewinae to status as a separate subfamily. This latter status was adopted by Dietrich (2005) in a key to cicadellid family-group taxa. Two other taxa most recently treated as separate subfamilies (Young 1986, Dietrich 2005), Makilingiinae Baker and Tinterominae Godoy & Webb, inhabit similar wet tropical montane forest habitats and resemble Mileewinae in having dorsal ocelli, reduced forewing venation, and also the hind wing submarginal vein very close to the wing margin distally (Dietrich 2005). Both are currently known only based on their type genera: *Makilingia* Baker, previously known only from the Philippines but recently recorded from Thailand (Dietrich, unpublished); and *Tinteromus* Godoy & Webb, previously known only from Costa Rica, but recently recorded from Colombia and Peru (D. M. Takiya, unpublished).

Phylogenetic analyses of both morphological and molecular data (Dietrich 1999, 2004, Dietrich et al. 2001, 2005, 2010) have consistently placed Mileewinae, Makilingiinae, and Tinterominae in a well-supported clade also comprising subfamilies Evacanthinae (sensu Dietrich 2004), Cicadellinae (sensu Young 1968), Signoretiinae (sensu Dietrich 2005), and Typhlocybinae but have not adequately resolved the relationships among major lineages within this clade. However, because the first three mentioned taxa share a unique combination of morphological traits (see below), they may represent a monophyletic group. Thus, they are here treated as tribes of a single subfamily, Mileewinae (sensu lato).

In a recent taxonomic review and morphology-based phylogenetic analysis of Evacanthinae sensu lato (including tribes Balbillini, Evacanthini, Nirvanini, and Pagaroniini), Dietrich (2004) excluded the enigmatic Andean genus *Tungurahuala* Kramer from that subfamily, placing it in Cicadellinae based on the structure of the ovipositor. Further morphological study of *Tungurahuala* and several South American species representing a new genus (described below), suggests that these two genera are most closely related to Mileewinae. The unique features uniting these two genera and their lack of apparent close relationship to other known leafhoppers support their placement in a new tribe, described below.

## Methods

Morphological terminology follows Oman (1949) and Kramer (1950) except that groups of macrosetae on the legs are named using the system of Rakitov (1998) and terminology for nymphs follows Dmitriev (2002). Setal rows on the femur and tibia are referred to according to their position, assuming that the leg is fully extended perpendicular to the sagittal plane of the body, as follows: AD=anterodorsal; AM=anteromedial; AV=anteroventral, PD=posterodorsal; and PV=posteroventral. Individual setae are numbered sequentially, beginning with the most distal. Numerical formulae are used to indicate the numbers of setae on the pro- and mesothoracic tibiae and the metathoracic femur. For tibiae the formula given is AD+PD; for the femur, the formula follows standard practice in which, for example, 2+2+1 means that a distal pair, a penultimate pair, and a single antepenultimate seta are present. Measurements are given in millimeters for males and females, respectively; body length is measured from the apex of the head to the apex of the forewing in repose. Digital photographs were taken using a Q-Imaging Micropublisher digital camera mounted on a stereo or compound microscope.

Specimens examined are deposited in the following institutions: Humboldt Institute, Villa Leyva, Colombia (HIC); Illinois Natural History Survey (INHS); North Carolina State University, Raleigh (NCSU); and Universidad de San Marcos, Lima, Peru (USML).

## Results

### Subfamily Mileewinae

htp://species-id.net/wiki/Mileewinae

Mileewinae Evans, 1947
Makilingiinae Evans, 1947, syn. n.
Tinterominae Godoy & Webb, 1994, syn. n.

#### Redescription.

Head with lorum extended little or no farther dorsad than clypeal suture; gena partially or entirely concealing triangular proepisternum; anteclypeus strongly convex, tapered distally; frontoclypeus without median longitudinal carina; ocelli on crown anteromesad of eyes, well separated from anterior margin; crown glabrous or punctate, without oblique lateral submarginal carinae; antennal base near anterodorsal corner of eye. Forewing (Figs 19, 21, 23, 25, 27, 29) with crossvein s (= “r" of Evans 1947) absent (outer anteapical cell absent); inner apical cell elongate, more or less parallel-sided, extended to apical margin. Hind wing (Figs 20, 22, 24, 26, 28, 30) with submarginal vein very close to or coincident with edge of wing along apical and costal margins; R2+3 complete. Front femur (Figs 33, 34) row AV without enlarged basal setae; hind femoral setal formula 2+1+1; hind tarsomere I pecten with 4 or fewer setae, including 0–2 platellae. Male subgenital plates (Figs 37–45) usually elongate, depressed basally, expanded medially or distally in lateral view (triangular in Makilingiini), macrosetae when present well separated from lateral margin and scattered or uniseriate; style apex (Fig. 47) usually cheliform, apophysis usually with preapical tooth; connective Y- or T-shaped, usually with median anterior lobe. Female second valvulae (Figs 71, 75) with distal paired blades comprising 50% or more of entire length of ovipositor.

#### Notes.

Mileewinae, as here redefined, are most readily distinguished from other Cicadellidae by the following combination of features: head with ocelli on crown distant from eyes and margin, frontoclypeus without median longitudinal carina; forewing with only two anteapical cells; hind wing submarginal vein very close to margin at wing apex; female second valvulae with paired distal blades occupying 50% or more of their length. They key to couplet 46 in Dietrich's (2005) key to tribes of Cicadellidae, which comprises the three previously recognized taxa included here as tribes. Comparative study of morphological characters indicates that *Tungurahuala*, previously placed in Nirvaninae (Kramer 1965), and a related new genus, *Ilyapa*, are not closely related to other nirvanines and are more closely allied to Mileewinae (Dietrich 2004). Mileewinae previously included only the nominotypical tribe but is here redefined to include tribes Tinteromini and Makilingiini (both previously treated as separate subfamilies), as well as a new tribe, Tungurahualini, described below.

The hind wing venation is very similar among the tribes here included in Mileewinae, although the extant Old World genera currently included in Mileewini (*Mileewa* Distant, *Ujna* Distant, and *Processina* Yang, Deitz & Li ) share a unique pattern in which vein R2+3 is complete but does not extend to the wing apex (Fig. 22); thus in these three genera there appear to be only three closed apical cells reaching the wing apex instead of the usual four (Fig. 26). Interestingly, this pattern does not occur in the New World mileewine genus *Amahuaka* Melichar, or in the two genera of Mileewini described from Eocene Baltic amber (Gebicki and Szwedo 2001), all of which have vein R2+3 extended to the wing apex (Fig. 26). Gebicki and Szwedo (2001) suggested that, based on the wing venation, *Orsalebra robusta* Young belongs in Mileewinae; however, species of *Orsalebra* have the ocelli on the anterior margin of the crown and are similar to Alebrini (Typhlocybinae) in other respects, so Young's (1952) original placement in Alebrini appears to be correct. As noted by Takiya (2007), *Vidanoana* Young, a genus endemic to Chile and currently placed in Cicadellini has hind wing venation similar to that of Mileewinae (Young 1977). The placement of this genus needs to be re-evaluated through further comparative study beyond the scope of the present paper.

**Key to tribes of Mileewinae**

**Table:** 

1	Head with margin of crown sharply carinate and encroaching onto eye laterally (Figs 13, 16); pronotum distinctly punctate; front femur with setae AM1 and AV1 small or absent (Phillippines, Thailand)	Makilingiini
–	Head with margin of crown at most weakly carinate, not encroaching onto eye laterally (Figs 12, 17, 18); pronotum without distinct punctations or, if punctures present, then pronotum also strongly rugose (Figs 1–3); front femur with one or more enlarged ventral setae at or near apex (Figs 33–34)	2
2	Body strongly depressed (Figs 11–12), rostrum not exceeding front trochanters, frontoclypeus with distinct transverse ridge visible as shelf in lateral view (Figs 11–12); forewing crossvein r-m1 present (Figs 27, 29); hind tarsus much less than half length of tibia, tarsomere I without platellae (pale, baloonlike setae), apical pecten bearing spiniform macrosetae (Figs 35–36); female second valvulae (Figs 71, 75) with dorsal margin bearing numerous large teeth with smaller serrations between teeth (Neotropical region)	Tungurahualini
–	Body not depressed (Figs 16–18), rostrum extended well beyond front trochanters; frontoclypeus evenly convex or flat in profile without transverse ridge; forewing with or without crossvein r-m1 (Figs 21, 23); hind tarsus approximately half length of tibia or longer (Fig. 18), tarsomere I with apical pecten bearing one or more platellae; second valvulae with dorsal margin smooth or minutely serrate, without large teeth	3
3	Antennae longer than body; forewing crossvein r-m1 present, appendix absent (Fig. 23)	Tinteromini
–	Antennae shorter than body; forewing crossvein r-m1 absent, appendix well developed (Figs 21, 25)	Mileewini

**Figures 1–18.**
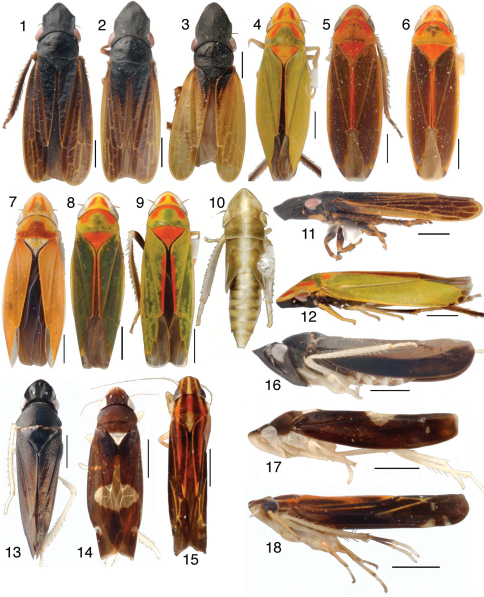
Mileewinae, scale bars = 1 mm **1–10** Tungurahualini, dorsal habitus **1** *Tungurahuala basilisca*, male from Colombia **2** *Tungurahuala acuminata*, male **3** same, female **4** *Ilyapa bifida*, male **5** *Ilyapa loca*, male **6** *Ilyapa longispina*, male **7** *Ilyapa ochrescens*, male **8** *Ilyapa recurvata*, male **9** *Ilyapa viridis*, male **10** same, 5th instar nymph **11–12** Tungurahualini, lateral habitus **11** *Tungurahuala acuminata*, male **12** *Ilyapa viridis*, male **13–18** Other Mileewinae **13** *Makilingia* sp., male from Thailand, dorsal habitus **14** same, *Mileewa margheritae*, male **15** same, *Tinteromus* sp., male from Colombia (full length of antenna not shown) **16** *Makilingia* sp., lateral habitus **17** same, *Mileewa margheritae* **18** same, *Tinteromus* sp.

### Tungurahualini trib. n.

urn:lsid:zoobank.org:act:E466750A-38A1-4798-A4E4-4E169D37F6E8

htp://species-id.net/wiki/Tungurahualini

#### Type genus.

*Tungurahuala* Kramer

#### Description.

Medium-sized leafhoppers (~6–8 mm), body depressed, head produced, face horizontal in profile, antenna shorter than width of head, frontoclypeus with transverse carina forming distinct shelf in lateral view (Figs 11–12, 31–32); anteclypeus extended to or slightly beyond lower margin of gena; lorum with lateral margin not extended to lateral margin of gena; rostrum short, not surpassing front trochanters. Front femur (Figs 33–34) with AM1 and AV1 enlarged, row AV with 0–1 preapical setae; intercalary row with 12 or more slender setae; hind tarsomere I without platellae, pecten with four tapered macrosetae. Forewing (Figs 27, 29) vein R with three branches; two r-m crossveins present; appendix absent or very narrow; hind wing vein R2+3 complete, extended to wing apex. Male pygofer (Fig. 37) with well developed ventral appendage, dorsal appendage absent, several macrosetae present distally; anal tube usually with paired ventrolateral processes; valve (Fig. 38) short, transverse, broadly fused to pygofer; subgenital plate broadest near base in ventral view, expanded medially in lateral view, with numerous scattered stout submarginal setae; connective stem as long as or longer than arms; style (Figs 47–51) cheliform with preapical lobe greatly enlarged and preapical tooth distinct. Female first valvulae with dorsal sculpturing strigate (Fig. 70) or concatenate (Fig. 73); second valvulae (Figs 71, 74) with small serrations between larger teeth.

#### Notes.

Tungurahualini resemble other Mileewinae in having the ocelli on the crown distant from the margin, the frontoclypeus and clypellus strongly convex, the forewing with the inner apical cell elongate and parallel-sided, the hind femur with macrosetal formula 2+1+1, and the second valvulae with the toothed distal blades longer than the basal fused section. They differ from other Mileewinae in having the head depressed with the face horizontal, the frontoclypeus with a transverse carina (Figs 31–32) forming a distinct shelf in profile, the first hind tarsomere pecten with spiniform setae (platellae absent), and the subgenital plate with numerous scattered macrosetae (see also Key).

A previous cladistic analysis (Dietrich 2004) consistently grouped the two genera included in Tungurahualini together in a clade also comprising the single included representative of Makilingiini. A more recent morphology-based analysis of the entire family Cicadellidae (Dietrich et al. 2010) placed Tungurahualini within a paraphyletic assemblage, comprising the other tribes here included in Mileewinae, that gave rise to Cicadellinae and Typhlocybinae. More detailed analysis of this entire lineage will be needed to further elucidate the phylogenetic status of Mileewinae and its included tribes.

Tungurahualini resemble Nirvanini (Evacanthinae, sensu Dietrich 2004) in having the body depressed with the head strongly produced and the face horizontal. The male genitalia also resemble those of Nirvanini, particularly the structure of the style (apex foot-shaped) and aedeagus (base with paired dorsal processes). However, unlike Nirvanini (and other Evacanthinae), the crown of the new tribe lacks a distinct marginal carina, the ocelli are distant from the margin, the face lacks a median longitudinal carina, crossvein r-m1 is present in the forewing, and the front femur lacks enlarged basal setae in row AV. The presence of spiniform setae rather than platellae at the apex of the first hind tarsomere is an unusual trait shared with some Oriental Evacanthini, but evacanthines differ in having the crown distinctly elevated mesad of the eyes, with marginal and submarginal carinae, and ocelli near the crown margin.

Species of Tungurahualini are presently known only from cloud forests in the northern and central Andean regions of the New World tropics.

**Key to genera of Tungurahualini**

**Table:** 

1	Head and pronotum uniformly black dorsally; crown margin pentagonal in dorsal view	*Tungurahuala* Kramer
–	Head and pronotum pale orange, white or green with distinct reddish orange markings; crown margin parabolic in dorsal view	*Ilyapa* gen. n.

### Tungurahuala

Kramer

htp://species-id.net/wiki/Tungurahuala

Tungurahuala Kramer 1965: 68. Type species *Tungurahuala basiliscus* Kramer by original designation. New placement.

#### Redescription.

Elongate, strongly depressed, leafhoppers (Figs 1–3, 11). Coloration dark brown to black; face with dull yellow band extended from lora across base of clypellus and apex of clypeus. Crown unevenly convex, coarsely granulose and densely clothed with minute setae, pentagonal in dorsal view; marginal carina present apically, becoming obsolete posterolaterally; median longitudinal carina weakly delimited; ocelli on crown anterad of eyes, slightly closer to lateral margin than to midline; antennal ledge broad, depressed, coincident with lateral margin of crown; flagellum slightly shorter than crown width; mesal margin of eye entire; lateral frontal suture absent dorsad of antennal ledge; frontoclypeus (Fig. 31) rugulose medially with well developed muscle scars laterally, oblique anteroventral section separated from nearly horizontal posteroventral section by transverse ridge; transclypeal suture indistinct; anteclypeus tapered, weakly convex, apex wider than lorum; lorum well separated from ventral genal margin; gena angulately produced, largely concealing proepisternum. Pronotum depressed, rugulose and minutely setose, narrower than head, lateral margins strongly carinate, carina even with eye, margins subparallel in dorsal view; exposed part of mesonotum and scutellum together wider than long. Forewing (Fig. 27) opaque basally, gradually becoming translucent distally; veins raised and well delimited, with marginal setae; costal flange well developed basally; R three-branched (rarely with 1–2 supranumerary branches), branches not reflexed; crossvein s absent (outer anteapical cell open distally); two r-m and three m-cu crossveins present; inner apical cell narrow; appendix absent. Hind wing (Fig. 28) with cell distad of r-m crossvein broadened distally. Prothoracic femur (Fig. 33) stout, AM1 and AV1 well developed, intercalary row with ~13 close-set preapical setae; tibia short, weakly expanded distally, dorsal rows with few indistinct, widely spaced setae, AV well developed, PV with few scattered setae. Mesothoracic femur longer and wider than prothoracic femur, compressed, AV and PV each with several irregular setae, tibial rows with numerous poorly differentiated setae. Metathoracic tibia row AV with setae evenly spaced from base to apex; tarsomere I with several scattered plantar setae. Male sternum III apodemes well developed; pygofer (Fig. 37) short with scattered macrosetae dorsolaterally, with long, slender process densely clothed with minute spicules arising from ventrolateral margin and extending mesad into genital capsule, then dorsad; anal tube well sclerotized, broader than long in dorsal view, venter flat; valve (Fig. 38) short, straplike, narrowly fused to pygofer; plates triangular, depressed, extended well beyond posterior margin of pygofer, with lateral band and irregular submedial row of macrosetae, dorsolateral margin weakly sinuate in lateral view, base weakly constricted in ventral view; aedeagus (Figs 46–52) in lateral view with shaft split into dorsal gonopore-bearing section and tapered ventral process; connective (Figs 47–51) trilobed basally; style with large preapical lobe and attenuated, hooked apex. Female sternite VII (Fig. 65) subtruncate, concealing base of ovipositor; first valvulae (Fig. 69) slender, with dorsal and ventral preapical sculpturing irregularly strigate; second valvulae (Fig. 71) with basal fused area short, distal blades large, dorsal margin ascending in straight line, then gradually descending toward apex, declivous portion with ~7 widely spaced conical teeth and intervening serrations, apical fourth serrate, without teeth; third valvulae without macrosetae. Nymph unknown.

#### Notes.

Kramer (1965) described *Tungurahuala* based on a single male specimen of *Tungurahuala basiliscus* Kramer from Baños, Tungurahua, Ecuador, placing it in Nirvaninae but noting that it is “vastly different from any previously described" genus. Recent Malaise trap sampling in Colombia has yielded additional specimens of the type species and a new, closely related species, including the first known female specimen of the genus. The genus may be distinguished from its only known relative, *Ilyapa*,by the characters noted in the key.

**Key to species of *Tungurahuala* (males)**

**Table:** 

1	Anal tube without distinct apical ventrolateral spines; aedeagal shaft (Figs 46, 48) broad in lateral view, ventral process with dorsolateral lobes	*Tungurahuala basilisca* Kramer
–	Anal tube with pair of distinct apical ventrolateral spines (Fig. 37); aedeagal shaft (Fig. 50) narrow in lateral view, ventral process without dorsolateral lobes	*Tungurahuala acuminata*, sp. n.

### Tungurahuala basilisca

Kramer

htp://species-id.net/wiki/Tungurahuala_basilisca

Figs 1 46–49

Tungurahuala basiliscus Kramer 1965: 68 [incorrect original spelling]

#### Redescription.

Length male 7.6–7.9. Head (male) approximately ¼ length of forewing. Forewing dark brown to black. Male anal tube with apical ventrolateral spines very weakly developed or absent. Aedeagus with gonopore-bearing shaft in lateral view broad, apex obliquely truncate, posteroapical margin concave; ventral process with pair of microtrichiate dorsolateral lobes, in ventral view abruptly expanded preapically, apex broadly bilobed, in lateral view with apex convergent toward shaft and with dorsal margin entire.

#### Material examined.

Holotype male: ECUADOR: Mt. Tungurahua, Baños, 2500m, August 20, 1937 (W. Clarke-Macintyre) [USNM]. Other material: 1 male, COLOMBIA, Cundinamarca, PNN Chingaza Charrascales, 4°31'N, 73°45'W, 2990m, Malaise, 4–18 October 2001 (L. Cifuentes), M.2551 [HIC]; 1 male, Cundinamarca, PNN Chingaza Alto de la Bandera, 4°31'N, 73°45'W, 3660m, Malaise, 30 March -12 April 2001 (L. Cifuentes), M.1585 [HIC]; 1 male, same data except 27 December 2001–11 January 2002 (E. Raigoso), M.3023 [INHS].

#### Notes.

The specimens examined from Colombia are here considered conspecific with the holotype from Ecuador, although there is slight variation among specimens in size, coloration, and the shape of the aedeagus. Given the small number of specimens available, it seems prudent to consider these minor variations to be intra-specific, despite the considerable geographic disjunction among the known populations.

Although Kramer (1965) did not explicitly indicate the gender of the name *Tungurahuala*, it is here interpreted as feminine due to its ending (ICZN Art. 30.2.4). Thus, Kramer's original spelling of the species name, which has a masculine ending, is incorrect and the spelling here emended to agree in gender with the genus name.

**Figures 19–30.**
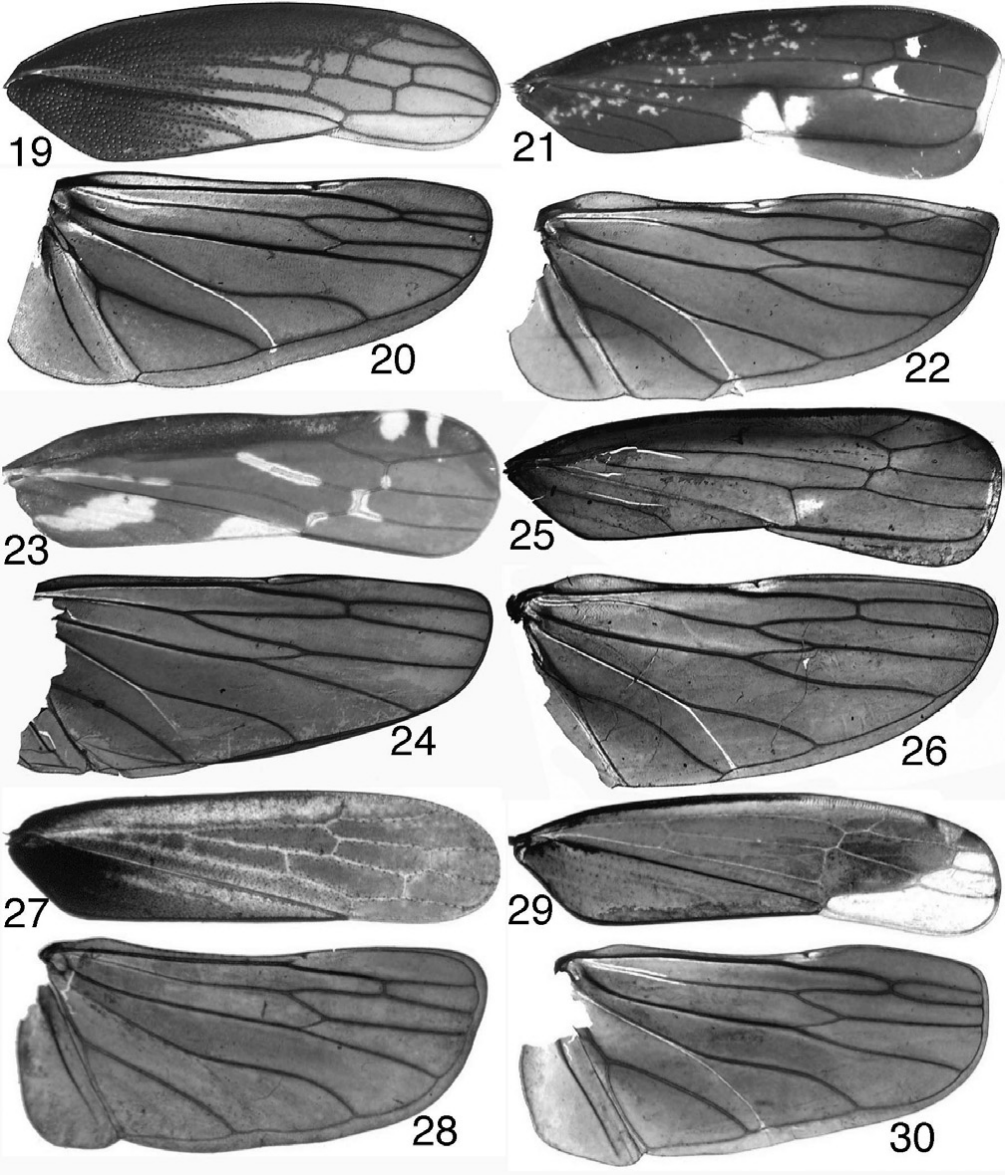
Mileewinae, wings **19–20** *Makilingia* sp. (Thailand), fore- and hind wing **21–22** same, *Mileewa margheritae* **23–24** same, *Tinteromus* sp. (Colombia) **25–26** same, *Amahuaka* sp. (Mexico) **27–28** *Tungurahuala acuminata* **29–30** same, *Ilyapa viridis*.

**Figures 31–45.**
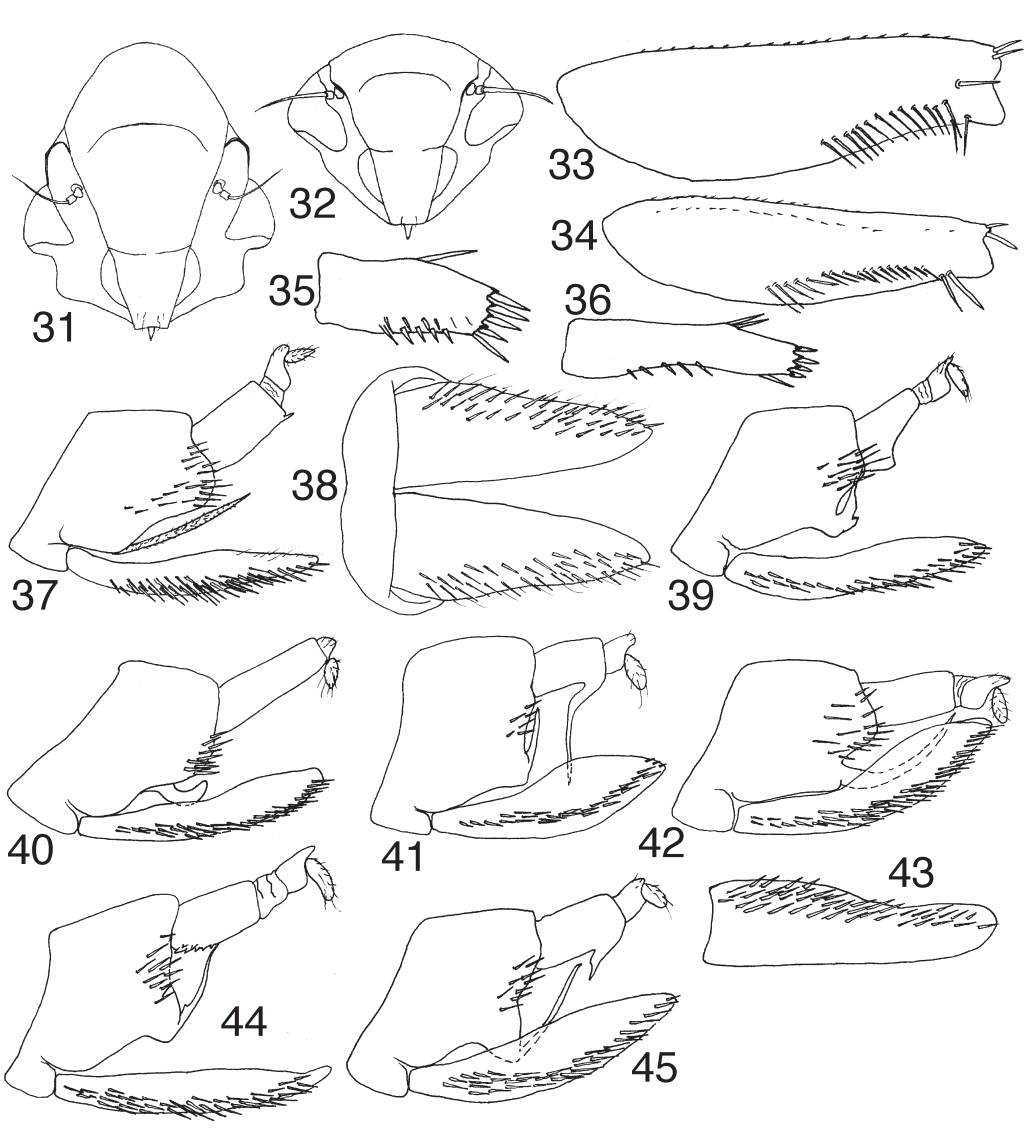
Tungurahualini **31–32** head, anteroventral view **31** *Tungurahuala acuminata* **32** *Ilyapa viridis* **33–34** prothoracic femur, anterior view **33** *Tungurahuala acuminata* **34** *Ilyapa viridis* **35–36** hind tarsomere I, ventral view **35** *Tungurahuala acuminata* **36** *Ilyapa viridis* **37** *Tungurahuala acuminata*, genital capsule, lateral view **38** same, valve and subgenital plates, ventral view **39** *Ilyapa bifida*, genital capsule, lateral view **40** same, *Ilyapa loca* **41** same, *Ilyapa longispina* **42** same, *Ilyapa ochrescens* **43** *Ilyapa ochrescens*, left subgenital plate, ventral view **44** *Ilyapa recurvata*, genital capsule, lateral view **45** same, *Ilyapa viridis*.

### Tungurahuala acuminata sp. n.

urn:lsid:zoobank.org:act:FD180D56-E0FC-4CCC-9C28-4F4C8FB090FE

htp://species-id.net/wiki/Tungurahuala_acuminata

Figs 2, 3, 11 27, 28 31, 33, 35, 37, 38 50–52 65, 69–71

#### Description.

Length male 7.5–7.6 mm, female 7.6 mm. Head (male) approximately 1/3 as long as forewing (female with head proportionately longer compared to forewing). Forewing dark brown with tan pigment along costal area, mostly tan in female. Male anal tube with apical ventrolateral spine well developed. Aedeagus with gonopore-bearing shaft slender, apex rounded; ventral process acuminate, without dorsolateral lobes, apex in ventral view narrower than shaft.

#### Material examined.

Holotype male: COLOMBIA, Boyacá, SFF Iguaque Lagunillas, 5°25'N, 73°27'W, 3380m, Malaise 2–18 May 2001 (P. Reina), M.1756. Paratypes: 1 male, same data except 28 June–19 July 2001, M.1966 [HIC]; 1 male, Boyacá, SFF Iguaque Qda. Carrizal, 5°25'N, 73°27'W, 3350m, Malaise 13 – 30 July 2000 (P. Reina), M.379; 1 male, same data except 21 January–9 February 2001; 1 male and 1 female, Boyacá, SFF Iguaque Cabaña Mamarramos, 5°25'N, 73°27'W, 2855m, Malaise 7–21 January 2001 (P. Reina), M.1252; 1 male, same data except 21 December 2000–7 January 2001, M.1072 [HIC, INHS].

#### Etymology.

The name refers to the acuminate ventral process of the aedeagus.

#### Notes.

This species resembles *Tungurahuala basilisca* in overall structure, but the head is proportionately longer and the coloration of the forewing is lighter overall, although variable among specimens examined.

### Ilyapa gen. n.

urn:lsid:zoobank.org:act:7A6B2217-9F1E-4D41-B680-BFD78F3FE553

htp://species-id.net/wiki/Ilyapa

#### Type species.

*Ilyapa longispina*, sp. n.

#### Description.

Medium sized, depressed leafhoppers (Figs 4–10, 12). Coloration pale orange or green with red/orange markings dorsally; crown with pair of oblique red maculae mesad of ocelli, pronotum with semicircular orange macula anteriorly, face and thoracic sternites black, legs dark basally and pale distally, abdomen heavily marked with dark brown dorsally and with varying amounts of dark brown pigmentation ventrally. Crown depressed, finely granulose, without setae, weakly pentagonal in dorsal view, marginal and medial carinae absent; ocelli on crown anterad of eyes slightly closer to lateral margin than to midline; antennal ledge broadly depressed, coincident with crown margin; flagellum slightly shorter than crown width; mesal margin of eye emarginate; frontoclypeus granulose with oblique anterodorsal section separated from nearly horizontal posteroventral section by distinct transverse ridge; muscle scars distinct laterally; clypeal suture obsolete medially; anteclypeus convex, tapered, apex narrower than lorum; lorum well separated from ventral genal margin; gena weakly produced laterally, partially concealing proepisternum. Pronotum weakly convex with irregular transverse striations, lateral margins divergent posterad, slightly wider than head, strongly carinate, carina even with eye. Exposed part of mesonotum and scutellum wider than long. Forewing (Fig. 29) opaquely sclerotized except apical cells, veins distinct but without marginal setae; R three branched, crossvein s absent; two r-m and 3–4 m-cu crossveins present; apical cells 2 and 3 very short; inner apical cell relatively broad; appendix very narrow. Hind wing (Fig. 30) with cell distad of r-m crossvein parallel-sided or narrowed distally. Prothoracic femur (Fig. 34) slender, AM1 large, located on ventral margin, AV1 well developed, row AV without preapical setae; intercalary row with ~17 slender, close-set setae; tibia 1+1, PV absent. Mesothoracic femur equal in length but wider than prothoracic femur; AV and PV with few widely separated setae, tibial row PD with apical seta, other rows with few irregularly spaced setae. Metathoracic femur macrosetal formula 2+1+1, rarely 2+1+1+1, tibia and tarsus as in *Tungurahuala*. Male with apodemes of sternite III well developed; pygofer (Figs 39–45) short with scattered macrosetae dorsolaterally, with ventral process glabrous, slender, arising posteroventrally and curved posterodorsad; anal tube in dorsal view as long as broad, flat ventrally, with or without pair of ventrolateral processes distally; valve short, rectangular, narrowly fused to pygofer; plates depressed basally, expanded and slightly compressed distally with band of macrosetae extended from lateral margin of base posteriorly across middle of apex; aedeagal shaft (Figs 53–62) arcuate posteriorly, often asymmetrical, gonopore apical; connective trilobed anteriorly, stem broad and depressed; style sinuate with large, sparsely setose preapical lobe, apophysis acuminate with ventral preapical tooth. Female seventh sternite (Figs 65–68) longer than sixth and concealing basal half of ovipositor in repose, posterior margin produced; first valvulae (Fig. 72–73) slender, dorsal sculpturing concatenate, second valvulae (Fig. 74) similar to those of *Tungurahuala* but with dorsal teeth more numerous, prominent and closely spaced, and dorsum at base of blade evenly rounded rather than angulate. Fifth instar nymph (Fig. 10) with overall form and chaetotaxy similar to that of adult except coloration pale greenish yellow with median dorsal longitudinal white stripe; crown with acrometope well delimited, longer than wide; metope well delimited; ocellar precursors well delimited and positioned as in adult on coryphe anteromesad of eyes, well separated from margin; face with distinct transverse shelf corresponding to epistomal suture (as in adult), cibarial muscle scars distinct; dorsum glabrous with scattered sparse, minute setae; enlarged setae absent; hind tarsomere I with three apical platellae.

#### Etymology.

The name *Ilyapa* is based on that of the Inca god of thunder, lightning, and rain, but the gender is here considered feminine due to its ending.

#### Notes.

This genus is closely related to *Tungurahuala*, as indicated by the similarities in cephalic structure (ocelli distant from margin, frontoclypeus with transverse carina), forewing venation (crossvein s lacking), leg chaetotaxy (hind tarsomere I pecten with tapered macrosetae), and male genitalia (pygofer with recurved posteroventral process, style with strong preapical lobe). It differs from *Tungurahuala* in the characters noted in the key.

The genus is described based on six species from the Andean region of South America. The species inhabit cloud forests and have been collected by sweeping grasses and other herbaceous vegetation in the understory. They are readily distinguished by differences in coloration, head proportions, and the structure of the male genitalia.

**Key to species of *Ilyapa* (males)**

**Table:** 

1	Aedeagal shaft (Figs 59–60) bilaterally symmetrical, compressed and broad in lateral view	*Ilyapa ochrescens*, sp. n.
–	Aedeagal shaft strongly asymmetrical, elongate, slender and tubular	2
2	Aedeagus (Figs 55–56) with depressed, apically truncate ventral process arising near base; gonopore-bearing shaft elongate, spinelike, evenly tapered distally, without process	*Ilyapa loca*, sp. n.
–	Aedeagus without basal process, shaft with one or more distal processes	3
3	Aedeagus (Figs 58, 64) with distal process extended laterad at approximately right angle in ventral view	4
–	Aedeagus with distal process not extended laterad at right angle, either strongly recurved ventrad or more or less continuing in line with shaft	5
4	Distal process of aedeagus bifid (Fig. 64)	*Ilyapa viridis*, sp. n.
–	Distal process of aedeagus unbranched (Fig. 58)	*Ilyapa longispina*, sp. n.
5	Aedeagus (Fig. 61) with single distal process strongly recurved ventrad, apex branched; anal tube with several small, irregular ventrolateral teeth (Fig. 44)	*Ilyapa recurvata*, sp. n.
–	Aedeagus (Figs 53–54) with two distal processes more or less aligned with shaft, asymmetrically curved, one process with angulate projection near base; anal tube with pair of short triangular projections near base (Fig. 39)	*Ilyapa bifida*, sp. n.

### Ilyapa bifida sp. n.

urn:lsid:zoobank.org:act:26BD4DCA-8225-43E5-AD09-1D484CC59C40

htp://species-id.net/wiki/Ilyapa_bifida

Figs 4, 12 39 53, 54

#### Description.

Length male 6.6–6.9 mm, female 7.0 mm. Crown pale orange-yellow, orange-red maculae broad, overlapping ocelli, anterior margin forming acute angle; pronotum and opaque areas of forewing bright green (mottled with yellow in specimens removed from ethanol), pronotum with semicircular macula distinct. Male pygofer processes extended mesad and curved dorsad but not or only slightly crossing midline; anal tube process short, triangular. Aedeagus asymmetrical, shaft tubular, in lateral view V-shaped basally, arched and sinuate distally, terminating in two slender, bladelike processes continuing in line with shaft but asymmetrically curved, one process with angulate projection near base. Female seventh sternite with posterior margin slightly produced, rounded medially. Fifth instar nymph pale olive green dorsally with broad white median longitidinal stripe extended entire length of body; venter white.

#### Material examined.

Holotype male: PERU, Pasco, Yanachaga-Chemillén N.P., Refugio El Cedro, 2420 m, 10°33'07"S, 75°21'27"W, 10 October 2002 (D. M. Takiya) PE07 [USML]. Paratypes: 1 male, same data except 12 October 2002 (R. Rakitov) [INHS].

#### Etymology.

The species name refers to the bifid apex of the aedeagus.

#### Notes.

This species may be distinguished by its relatively long crown and the bifid apex of the aedeagus.

### Ilyapa loca sp. n.

urn:lsid:zoobank.org:act:8E950B3B-7F16-47E6-96F5-C5729EA156C0

htp://species-id.net/wiki/Ilyapa_loca

Figs 5 40 55–56

#### Description.

Length male 6.1 mm. Coloration as described for *Ilyapa bifida* except crown margin white, orange-red maculae broader, and pronotum almost entirely orange; apical margin of crown forming obtuse angle. Male pygofer processes short, broad, and digitiform; anal tube without processes. Aedeagus highly asymmetrical; gonopore-bearing shaft acuminate, extended to left posterodorsad and gradually curved mesad, apex without process; ventral process depressed, apex expanded, truncate, and even with style apices. Female unknown.

#### Material examined.

Holotype male, PERU, Chanchamayo, 25 July 1960 (Young and Ramirez) [NCSU].

#### Etymology.

The species name means “crazy" and refers to the bizarre, asymmetrical aedeagus.

#### Notes.

This species may be distinguished by its relatively short crown and by the presence of a long, unpaired ventral process arising from the base of the aedeagal shaft.

### Ilyapa longispina sp. n.

urn:lsid:zoobank.org:act:00015CA5-8E24-4357-A657-7B8D3B5F6638

htp://species-id.net/wiki/Ilyapa_longispina

Figs 6 41 57–58 67, 72–74

#### Description.

Length male 5.9–6.2 mm; female 7.1–7.5 mm. Coloration as described for *Ilyapa bifida* except crown margin mostly white; apical margin of crown forming approximately right angle. Male pygofer processes slender, crossing posteromedially; anal tube with pair of retrorse posterolateral spines; plate apex angulate mesally. Anal tube processes long with apices curved ventrad; pygofer processes shorter, not meeting medially; apical process of aedeagus more elongate, with minute preapical spine posteriorly. Female seventh sternite with posterior margin trilobed with median lobe acute and larger than lateral lobes.

#### Material examined.

Holotype male: PERU, Chanchamayo, 25 July 1960 (Salazar and Ramirez) [NCSU]. Paratypes: 1 male, same locality, 22 July 1960 (C. Ramirez) [INHS]; 3 females, same locality except 21 and 25 July 1960 [NCSU, INHS]. Other material: 3 males, PERU, Ucayali, 8 km E Abra La Divisoria, 1250 m, 9°9'57"S, 75°48'11"W, 25 October 2002 (R. A. Rakitov) sweeping, 02-39-2; 1 male, 1 female, PERU, Pasco, Yanachaga-Chemillén N.P., Puesto de Control Huampal, 1050 m, 10°1'09"S, 75°34'27"W, 8 October 2002 (R. A. Rakitov) on grass; 1 male, same data except Refugio El Cedro, 2420 m, 10°33'07"S, 75°21'27"W, 10 October 2002, D.M. Takiya, PE07; 1 female PERU, Junin, 1 km S Minapichita, 2100 m 11°6'1"S, 75°25'30"W, 19 October 2002 (C. H. Dietrich) sweeping, 02-20-1 [INHS].

#### Etymology.

The species name refers to the long distal spine of the aedeagus.

#### Notes.

This species may be distinguished by its moderately long crown and by the elongate, laterally directed distal spine of the aedeagus.

One specimen from Yanachaga-Chemillén National Park, Peru, has the distal spine of the aedeagus extended to the right, mirroring the condition found in other examined specimens of this species. Specimens examined from Yanachaga-Chemillén National Park have the anal tube processes considerably shorter than those in the type series from Chanchamayo, but such variation is here considered to be intraspecific. Based on the material available for study, this is the most widespread and common species of the genus.

### Ilyapa ochrescens sp. n.

urn:lsid:zoobank.org:act:1D932E94-61AA-400E-B8D3-40F45CEF0D3D

htp://species-id.net/wiki/Ilyapa_ochrescens

Figs 7 42 59–60

#### Description.

Length male 6.5–6.8 mm. Nearly uniform pale orange dorsally, orange-red maculae slender; apical margin of crown forming acute angle, pronotal macula indistinct. Male pygofer processes robust, not crossing posteromedially; anal tube without ventrolateral processes; aedeagal shaft symmetrical, pillarlike, compressed, in lateral view extended dorsad and bent posterad at right angle, with pair of acute anterodorsal processes extended anterad, posterodorsal extension with ventral margin irregular, apex expanded with ventral spine. Female unknown.

#### Material examined.

Holotype male: COLOMBIA, Cundinamarca, PNN Chingaza Valle Del Fraylejon, 4°31'N, 73°45'W, 3170m, Malaise 31 August–13 September 2000 (A. Pérez), M.732. Paratypes: 1 male, same coordinates, Chingaza Bosque Palacio, 2930m, Malaise 8–22 December 2000 (A. Cifuentes), M.1027; 1 male, same data except 3–16 March 2001(C. Vinchira and A. Cifuentes), M.1492 [HIC].

#### Etymology.

The species name refers to the mostly orange coloration of the dorsum.

#### Notes.

This species may be distinguished by its relatively long crown, predominantly orange coloration, and broad, strongly compressed aedeagal shaft.

### Ilyapa recurvata sp. n.

urn:lsid:zoobank.org:act:987F8A00-CC54-494D-AE56-7FDAD4F92AF3

htp://species-id.net/wiki/Ilyapa_recurvata

Figs 8 44 61–62

#### Description.

Length male 5.9 mm. External morphology and male terminalia similar to those of *Ilyapa longispina*, except as follows: anal tube without pair of ventrolateral processes but with several irregular teeth; aedeagus with distal process strongly recurved ventrad and anterad, branched near midlength with one branch about half length of other. Female unknown.

#### Material examined.

Holotype male: PERU, Huánuco, Carpish Pass, 2600 m, 9°43'3"S, 76°5'38"W, 27 October 2002 (C. H. Dietrich) sweeping, 02-45-1 [USML].

#### Etymology.

The species name refers to the strongly recurved apex of the aedeagus.

#### Notes.

This species may be distinguished by its relatively short crown and strongly recurved aedeagal apex.

### Ilyapa viridis sp. n.

urn:lsid:zoobank.org:act:D530E660-0B0C-4159-9EDF-4FD394B048C4

htp://species-id.net/wiki/Ilyapa_viridis

Figs 9 29–30 32, 34, 45 63–64 68

#### Description.

Length male 5.8–6.0, female 7.0 mm. Crown pale yellow medially, white laterally, orange/red maculae broad, overlapping ocelli; anterior margin forming approximately right angle; pronotum and opaque areas of forewing dark green (mottled with yellow in specimens removed from ethanol), pronotum with semicircular macula distinct. Male pygofer processes slender, crossing posteromedially; anal tube with pair of retrorse posterolateral spines. Aedeagus asymmetrical, shaft narrow, tubular and gradually tapered distally, in lateral view narrowly U-shaped with distal part attenuated and arcuate; apex with slender bifurcate process extended to left at right angle to shaft. Female seventh sternite posterior margin with small acute median tooth.

#### Material examined.

Holotype male: PERU, Pasco, Yanachaga-Chemillén N.P., 10°32'39.7"S, 75°22'00.1"W, 2300m, 10–13 October 2002 (D. Takiya, C. Peña, R. Rakitov) Malaise trap across Rio San Alberto [USML]. Paratypes: 1 male and 1 female, same data; 2 males, Yanachaga-Chemillén N.P., 10°32'S, 75°21'W, S. Alberto Valley ca. Refugio El Cedro, 2270–2420m, 12 October 2002 (R. Rakitov); 2 males, 1 female, same data except 10 October 2002 (D. M. Takiya); 8 females, same data except 12 October 2002 (D. M. Takiya) [INHS].

#### Etymology.

The species name refers to the mostly pale green coloration of the dorsum.

#### Notes.

This species closely resembles *Ilyapa longispina* in external morphology and in the male genitalia, but may be distinguished by the shorter, distinctly branched distal aedeagal process and by the distinctly smaller median lobe of female abdominal sternite VII.

In one examined male specimen, the configuration of the aedeagus is the mirror image of that of the other specimens.

**Figures 46–52.**
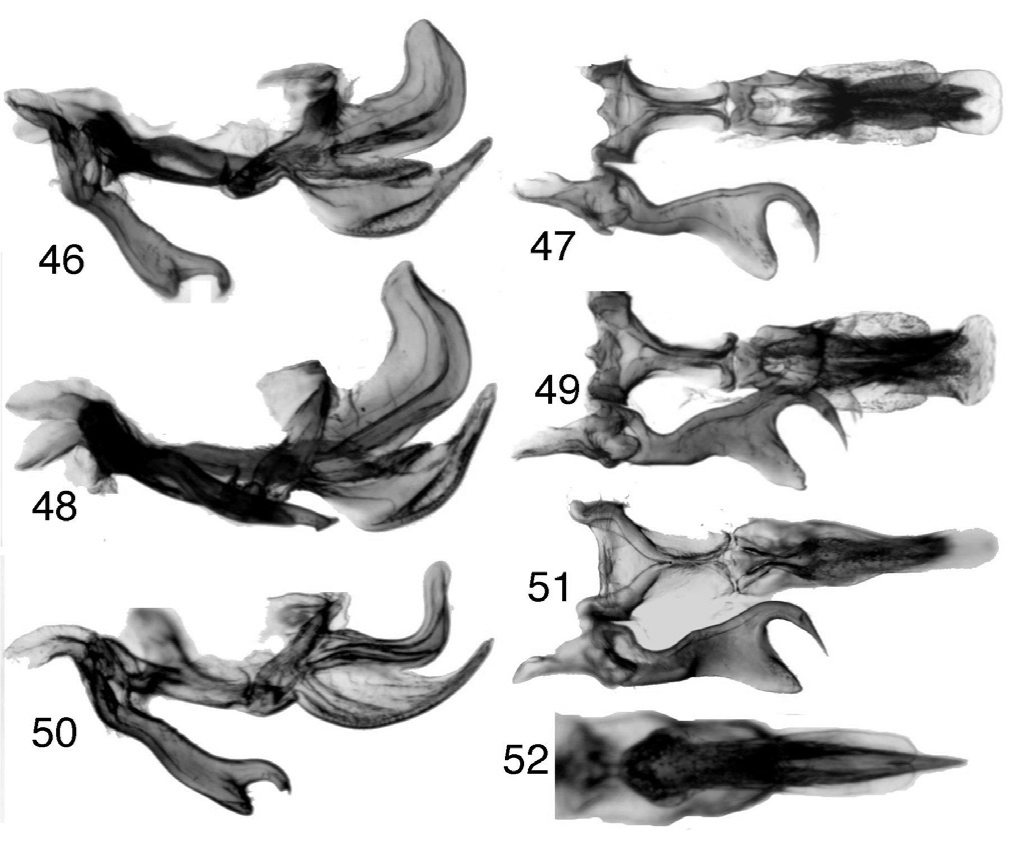
*Tungurahuala*, male genitalia **46** *Tungurahuala basilisca* (specimen from PNN Chingaza Alto de la Bandera, Cundinamarca, Colombia), genitalia, lateral view **47** same, ventral view (only right style shown) **48** *Tungurahuala basilisca* (specimen from PNN Chingaza Churrascales, Cundinamarca, Colombia), genitalia, lateral view **49** same, ventral view **50** *Tungurahuala acuminata*, genitalia, lateral view **51** same, ventral view **52** same, aedeagus, posteroventral view.

**Figures 53–64.**
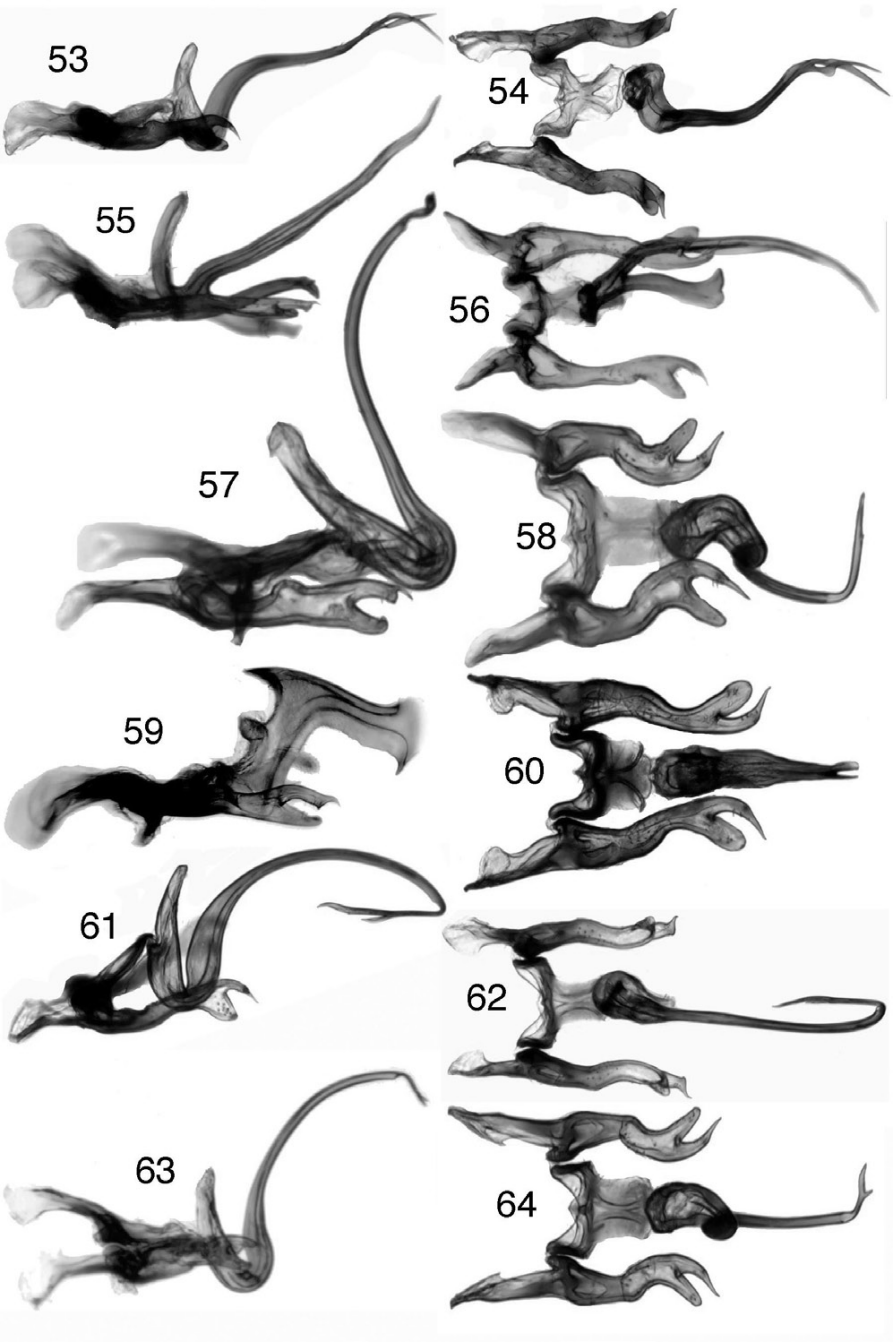
*Ilyapa*, male genitalia, lateral and ventral views **53–54** *Ilyapa bifida* **55–56** *Ilyapa loca* **57–58** *Ilyapa longispina* **59–60** *Ilyapa ochrescens* **61–62** *Ilyapa recurvata* **63–64** *Ilyapa viridis*.

**Figures 65–74.**
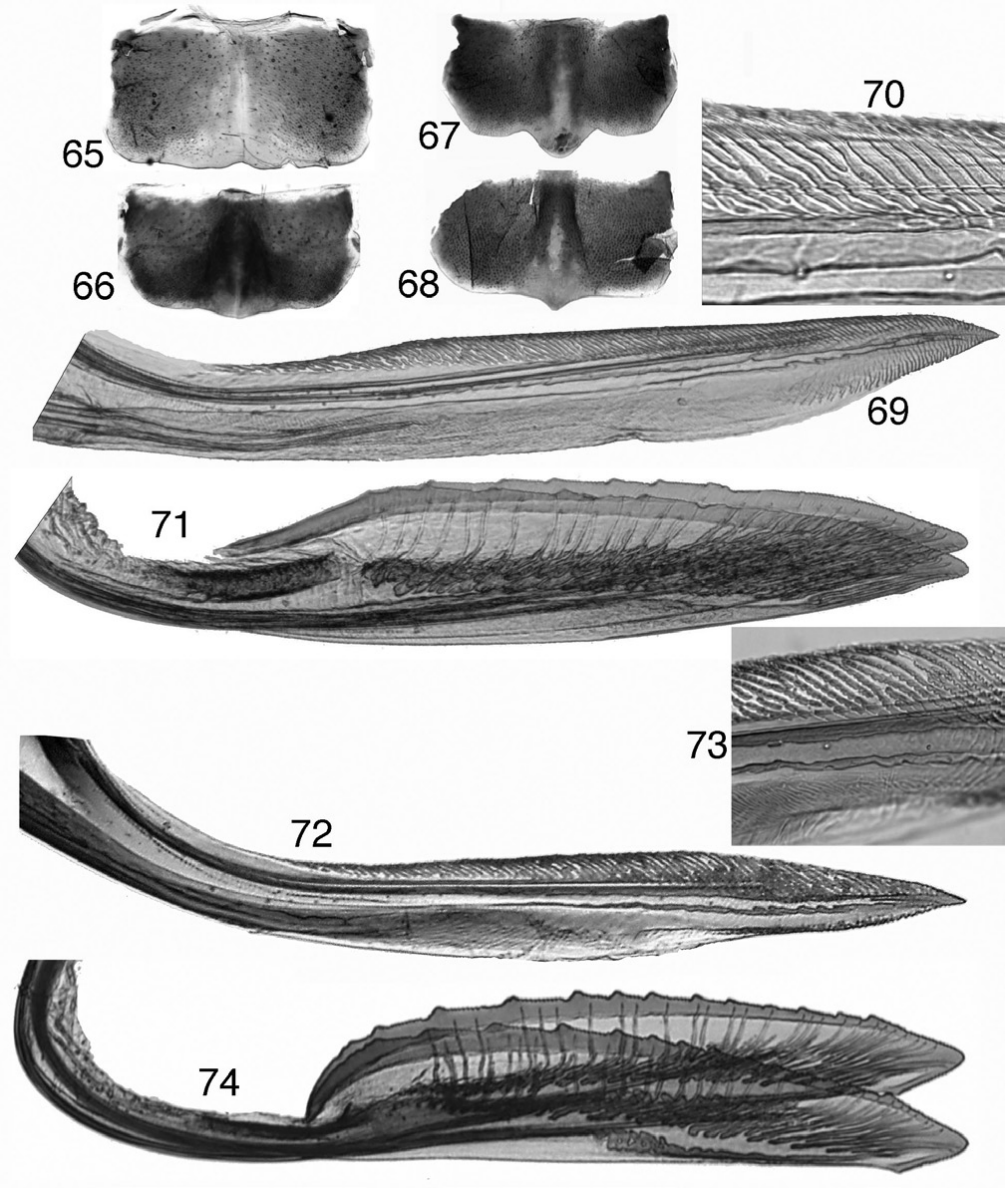
Tungurahualini, female terminalia **65–68** sternite VII **65** *Tungurahuala acuminata* **66** *Ilyapa bifida* **67** *Ilyapa longispina* **68** *Ilyapa viridis* **69–71** *Tungurahuala acuminata* **69** first valvula **70** same, detail of dorsal sculptured area **71** second valvulae **72–74** *Ilyapa longispina* **72** first valvula **73** same, detail of dorsal sculptured area **74** second valvulae

## Supplementary Material

XML Treatment for Mileewinae

XML Treatment for Tungurahualini

XML Treatment for Tungurahuala

XML Treatment for Tungurahuala basilisca

XML Treatment for Tungurahuala acuminata

XML Treatment for Ilyapa

XML Treatment for Ilyapa bifida

XML Treatment for Ilyapa loca

XML Treatment for Ilyapa longispina

XML Treatment for Ilyapa ochrescens

XML Treatment for Ilyapa recurvata

XML Treatment for Ilyapa viridis
